# Repellent efficacy of the nanogel containing *Acroptilon repens* essential oil in comparison with DEET against *Anopheles stephensi*

**DOI:** 10.1186/s13104-023-06538-1

**Published:** 2023-10-09

**Authors:** Elham Zarenezhad, Alireza Sanei-Dehkordi, Behina Babaalizadeh, Hajar Qasmei, Mahmoud Osanloo

**Affiliations:** 1 Noncommunicable Disease Research Center, Fasa University of Medical Sciences, Fasa, Iran; 2 Department of Biology and Control of Disease Vectors, School of Health, Hormozgan University of Medical Sciences, Bandar Abbas, Iran; 3 Infectious and Tropical Diseases Research Center, Hormozgan Health Institute, Hormozgan University of Medical Sciences, Bandar Abbas, Iran; 4 Department of Biochemistry, School of Medicine, Fasa University of Medical Sciences, Fasa, Iran; 5 Department of Medical Nanotechnology, School of Advanced Technologies in Medicine, Fasa University of Medical Sciences, Fasa, Iran

**Keywords:** DEET, Nanoemulsion, Mosquito, *Acroptilon repens*

## Abstract

**Objective:**

Malaria is a vector-borne disease that causes many deaths worldwide; repellents are a practical approach to malaria prevention, especially in endemic regions.

**Results:**

Gas chromatography-mass spectrometry analysis was used to identify compounds in *Acroptilon repens* essential oil (EO). Alpha-copaene (15.67%), α-cubenen (3.76%), caryophyllene oxide (14.00%), 1-heptadecane (5.61%), and δ-cadinene (2.84) were five major compounds. After that, the nanoemulsion containing the EO with a particle size of 46 ± 4 nm, SPAN 0.85, PDI 0.4, and zeta potential − 5.7 ± 0.4 mV was prepared. Then, it was gellified by adding CMC (carboxymethyl cellulose) to the nanoemulsion. Besides, ATR-FTIR analysis (Attenuated Total Reflection-Fourier Transform InfraRed) was used to confirm the EO’s successful loading in the nanogel. Finally, the protection time and repellent activity of nanogel compared to DEET (N, N-diethyl-meta-toluamide) were investigated against *Anopheles stephensi*. Interestingly, the nanogel with a protection time of 310 ± 45 min was significantly more potent than DEET (160 ± 17 min). It could thus be considered for future investigation against other mosquitoes.

**Supplementary Information:**

The online version contains supplementary material available at 10.1186/s13104-023-06538-1.

## Introduction

Malaria, with about half a million deaths annually, is one of the greatest health problems in the world, especially in developing countries [1]. Controlling mosquitoes is a practical strategy to reduce the transmission of this disease, especially in endemic regions; repellents are suggested [2]. However, excessive use of DEET as a gold-standard repellent causes mosquito resistance [3]. Moreover, routine exposure to DEET may induce neuronal degeneration in the brain [4]. Besides, it causes toxic reactions like cardiovascular and neurological side effects, allergy, and dermatitis [5]. Attempts to develop natural repellents have thus been increasing in recent years [6].

*Acroptilon repens* (Asteraceae), or *Rhaponticum repens*, is a widespread medicinal plant in Mongolia, Iran, Turkey, Armenia, the United States, and Canada. It has been used in traditional medicine as an emetic, antiepileptic, and anti-malaria [7]. In addition, its Essential oil (EO) polyphenol compounds are responsible for various biological effects such as antileishmanial, antioxidant, antibacterial, antimutagenic, anti-inflammatory, and antilarval activities [8, 9].

Furthermore, nanogels with high loading capacity, proper viscosity, biocompatibility, and biodegradability promise dosage forms for the stability improvement of EOs in topical drug delivery and repellents [10, 11]. To the best knowledge of the authors repellency effect of *A. repens* EO was not reported. So, in this study, the repellency effect of nanogel containing *A. repens* EO compared with DEET against *An. stephensi*.

## Main text

### Gas chromatography-mass spectrometry process

Whole plant-extracted *A. repens* EO was obtained from Zardband Pharmaceuticals Co (Iran). Agilent 6890 gas chromatography system was applied to identify the compounds of *A.repens* EO. The BPX5 column chromatography with 30 m length, 0.25 mm internal diameter, and 0.25 mm film thickness was used for separation. Firstly, the temperature was fixed at 50 °C for 5 min, then 3 °C /min to 240 °C, and finally held for 300 °C. For the injection port, the temperature was adjusted to 250 °C. Helium was applied as a carrier gas with a split flow of 35 mL/min with a septum purge of 5 mL/min. A mass spectrometer (model: Agilent 5973 N) was taken at 70 EV ionization energy with the Electron Impact Ionization method. The temperature of the ionization source was 553 °C, and full scan spectra at 40–500 were recorded. The chemstation software was used. The spectra were identified with the help of their inhibition index and compared with the index found in reference books and articles using the standard mass spectra of compounds and the information available in the computer library [12, 13]. The figure of recorded spectra is given in the S1 supplementary file.

### Preparation and characterizations of nanoemulsion and nanogel

Some of the compounds of *A. repens* EO are volatile; the spontaneous emulsification method was used to prepare oil in water nanoemulsion [14]. *A. repens* EO (2.4% w/v), tween 20 (6% w/v), and ethanol (1.6% w/v) was first stirred 10 min at 2000 rpm. Distilled water was then drowsily added to the final volume (20 mL) and stirred for 40 min to stabilize. After that, the prepared nanoemulsion was gellified by adding CMC (3.5% w/v); the mixture was stirred for 15 h at 2000 rpm. Besides, the blank gel was prepared similarly, without EO.

Dynamic light scattering apparatus equipped with a zeta sizer (Horiba Scientific SZ-100, Horiba, Japan) was used to investigate the nanoemulsion’s particle size, particle size, and zeta potential. Nanoemulsion with particle size < 200 nm, SPAN < 1, and PDI < 0.7 is considered proper size characteristics [15]. Besides, a rheometer machine (Anton Paar, MCR-302, Model MCR-302, Austria) was used to measure the viscosity of nanogel at different shear rates (0-100 s/1). The stability of nanogel was monitored for six months. The nanogel was incubated at room temperature and 4 °C and visually checked for biphasic and sedimentation.

ATR-FTIR was used for the investigation loading of the EO in the nanogel. The EO, blank gel, and nanogel were subjected to the device (ATR-FTIR Spectrometer, Model Tensor II, Bruker Company), and their spectra were recorded in 400 to 4000 cm^− 1^.

### Repellent bioassays

The used Bandar-e-Abbas strain of An. stephensi was supplied by Hormozgan University of Medical Science. Mosquitoes were reared, maintained, and tested (in a separate space or room) at 27 ± 2 ^o^C temperature, ≥ 70 ± 10% relative humidity, and a 12:12 h (light: dark) photoperiod. There were 250 adult female (5–7 days old), non-blood fed, and nulliparous mosquitoes in cages (40 × 40 × 40 cm). They were not fed for 14 h before repellency tests by removing 10% sugar solutions from the mosquito cage. A healthy 48-year-old male volunteer who did not smoke or drink alcohol and had no history of dermatological disease or allergic reactions to mosquito bites was selected to study the effectiveness of insect repellents. Before giving consent, the volunteer was interviewed and informed about the study objectives, methods, and possible discomforts caused by exposure to test substances and mosquito bites [16].

Samples were applied exclusively to the hairless region on the underside of the lower arm, spanning an 8 cm × 12.5 cm area, during individual bioassays. These samples consisted of 1 gram of nanogel, DEET (2.4%), or blank gel (see Figure S2 in supplementary file). Latex gloves covered the volunteer’s hand and placed it in the cage for 3 min. A 3-min test and 30-minute rest periods were totally continued until one landing and/or probing occurred in a 3-min test [16]. The bioassays were carried out in triplicates, and the results were presented as Mean ± SD. Besides, one-way ANOVA followed by the Tukey Post Hoc test was used to compare the efficacy of samples (IBM SPSS statistics 22 software, USA).

## Results

### **Ingredients of*Acroptilon repens’*EO**

Thirty-two compounds were identified in *A.repens* EO (Table 1). Five major compounds were α-Copaene (15.67%), α-Cubenen (3.76%), Caryophyllene oxide (14.00%), 1-Heptadecane (5.61%), and δ-Cadinene (2.84).

**Table 1:** Chemical ingredients identified in the *Acroptilon repens’* EO by gas chromatography-mass spectrometry

No	RT	Percent%	Component	KI	type
1	16.30	0.07	P-Cymene	1025	MH^1^
2	16.46	0.10	Limonene	1029	MH
3	25.57	0.10	Decanal	1202	MH
4	29.25	0.71	1-Tridence	1292	Other
5	31.82	3.76	α-Cubenen	1351	SH^2^
6	32.89	0.38	Cyclosativene	1371	SH
7	33.18	15.67	α-Copaene	1374	SH
8	33.51	0.90	Trans-β-damascenone	1384	MO^3^
9	33.69	2.11	β-Cubenen	1388	SH
10	34.44	0.39	Cyperene	1398	SH
11	35.13	2.12	β-Caryophyllene	1419	SH
12	35.42	0.99	γ-Muurolen	1479	SH
13	35.57	0.55	α-Bergamotene	1434	SH
14	35.93	0.08	α-selinene	1498	SH
15	36.34	0.66	Geranyl acetone	1455	MO
16	36.74	1.13	β-Santalene	1459	SH
17	37.46	0.25	γ-Muurolen	1479	SH
18	38.14	1.00	β-selinence	1490	SH
19	38.40	1.56	Germacrene D	1485	SH
20	39.23	2.84	δ-Cadinene	1523	SH
21	39.49	2.76	Calamenene	1532	SH
22	39.88	0.16	Cadinadiene-1,4	1539	SH
23	41.81	1.55	Spathulenol	1578	SO^4^
24	41.99	14.00	Caryophyllene oxide	1583	SO
25	42.22	1.47	β-Copaene-4α-ol	1595	SO
26	43.70	1.14	β-Guaiene	1667	SH
27	45.677	5.61	1-Heptadecene	1692	Other
28	49.461	0.17	Octadecane	1800	Other
29	51.044	2.62	Hexahydrofarensyl acetone	1864	Other
30	53.462	0.18	Fransyl acetone	1913	Other
31	53.896	0.10	Methyl hexadecanoate	1921	Other
32	56.096	0.07	Heneicosane	2100	Other
		65.20	Total identified		

1: Monoterpene Hydrocarbons, 2: Sesquiterpene Hydrocarbons, 3: Oxygenated Monoterpenes, and 4: Oxygenated Sesquiterpenes

### The prepared nanoemulsion-based nanogel

**Fig. 1:**
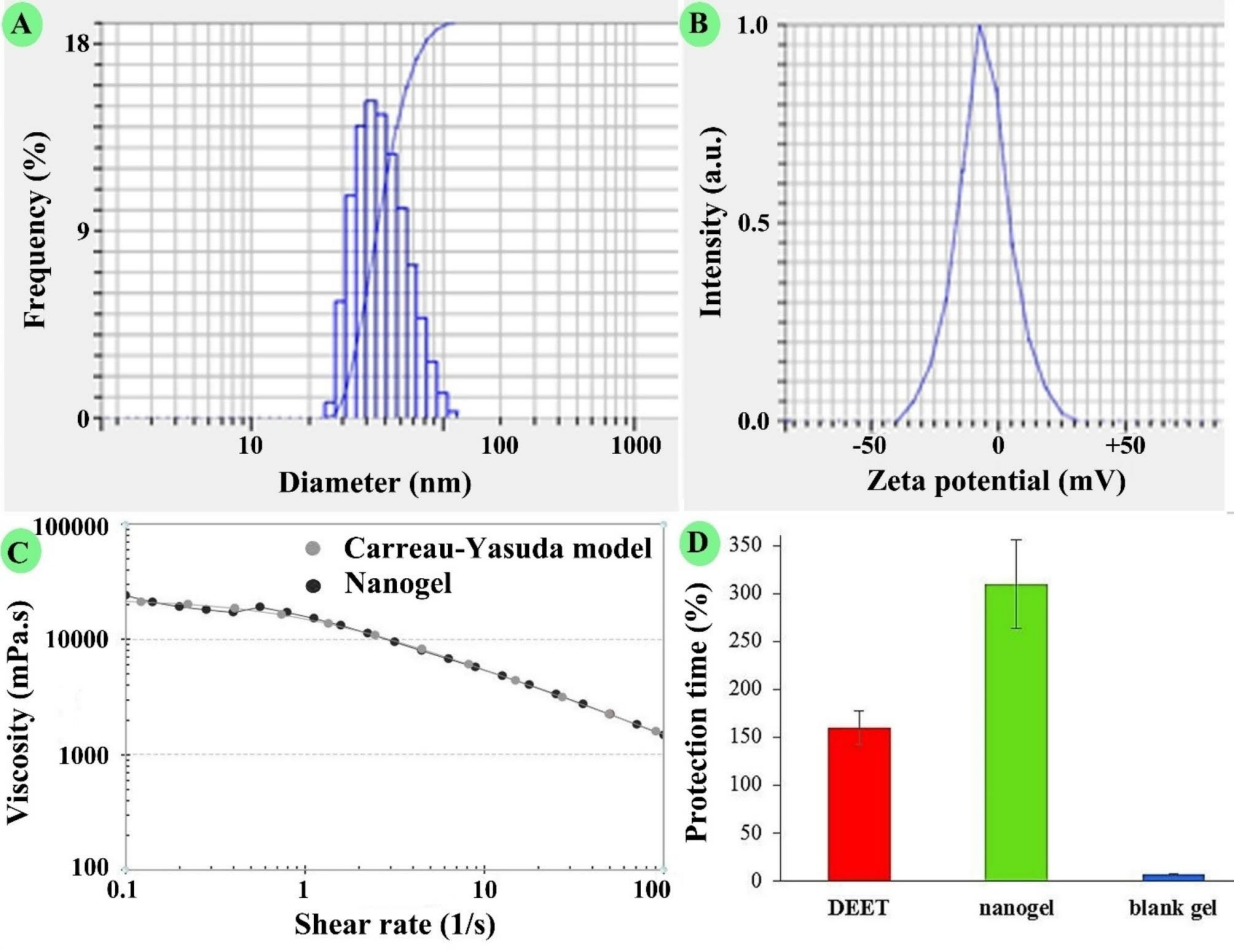
A: DLS analysis of the nanoemulsion containing *Acroptilon repens’* EO with a droplet size of 46 ± 4 nm, B: its zeta potential − 5.7 ± 0.4 mV, C: viscosity of the nanogel containing *A. repens* EO, and D: protection time of the nanogel against *Anopheles stephensi*

DLS result of the nanoemulsion with the particle size 46 ± 4 nm, SPAN 0.85, and PDI 0.4 is shown in Fig. 1A. As particle size is less than 200 nm, SPAN was less than 1, and PDI was less than 0.7; therefore, the nanoemulsion possesses proper size characteristics.

Furthermore, the potential zeta profile is shown in Fig. 1B; it was − 5.7 ± 0.4 mV. Besides, nanogel viscosity is shown in Fig. 1C; viscosity was investigated at share rates of 1 to 100 1/s. It was fitted with the Carreau-Yasuda standard model for non-Newtonian liquid [17].

### Successful loading of EO in nanogel

**Fig. 2:**
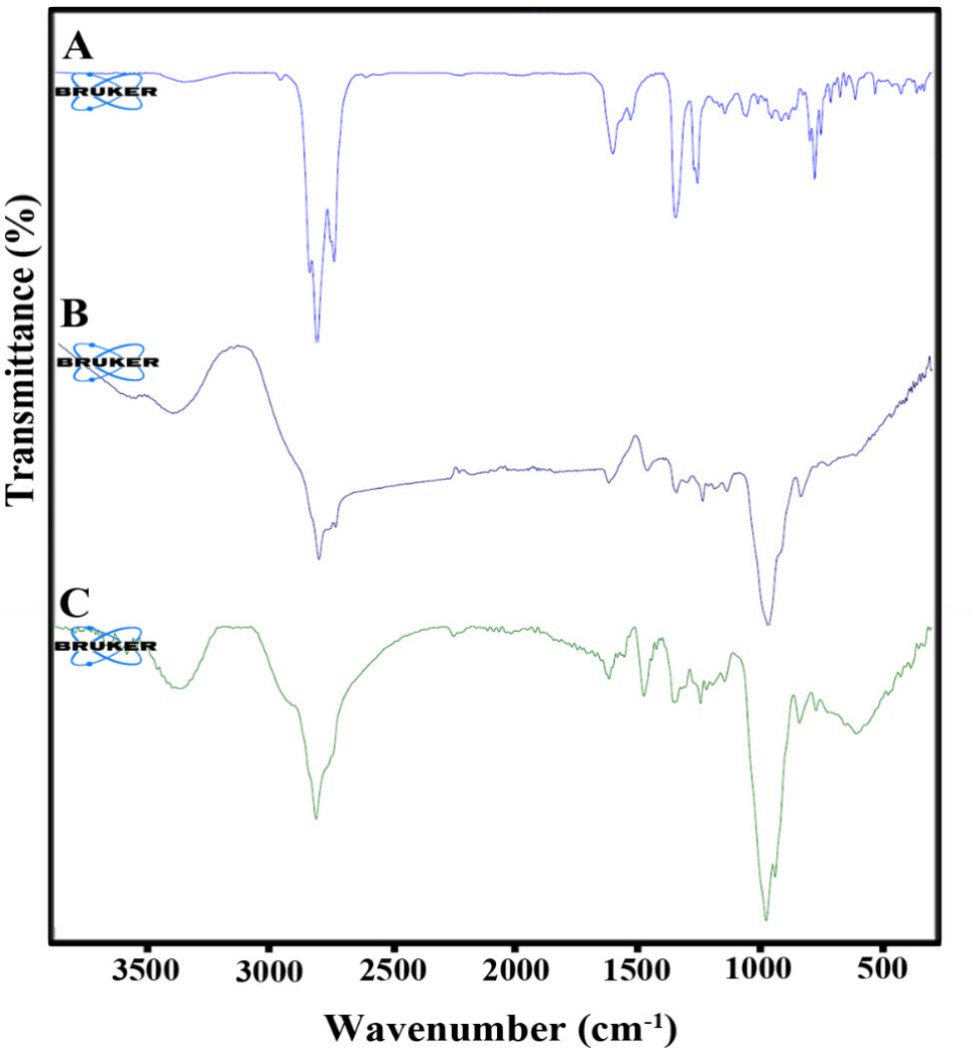
The ATR-FTIR spectra of A: *Acroptilon repens’* EO, B: blank gel, and C: nanogel

ATR-FTIR spectrum of *A. repens* EO is shown in Fig. (2 A). The broad bands at 3467 cm^− 1^ can be related to the stretching vibration of OH, the band at 3074 cm^− 1^ attributed to = C-H, and the bands at 2954, 2923, and 2854 cm^− 1^ showed a stretching vibration of –CH. The characteristic band at 1713 cm^− 1^ corresponds to the stretching vibration of a carbonyl group. The absorption band at 1641 cm^− 1^ is allocated to the C = C vibration. The characteristic peaks at 995 cm^− 1^ and 888 cm^− 1^ can be attributed to C-H bending absorption.

In the blank nanogel spectrum, as shown in Fig. (2B), the broad and characteristic peak at about 3200–3600 cm^− 1^ is attributed to OH stretching vibration due to hydrogen bonding. The bands at 2923 cm-^1^ and 2855 cm^− 1^ correspond to C-H stretching vibration. Besides, the bands at 1734 and 1577 cm^− 1^ can be related to the carbonyl and COO groups in tween 20. The characteristic band at 1417 cm^− 1^ displayed CH_2_ bending, and the sharp band at 1081 cm^− 1^ is attributed to C-O stretching.

The nanogel spectrum is shown in Fig. 2C. The broad peak at about 3300–3700 cm^− 1^ is related to –OH stretching vibration. The peak at 2925 cm^− 1^ is attributed to C-H stretching in the EO, tween 20, and CMC. Besides, The band at 1726 cm^− 1^ can be assigned to the carbonyl group. The band at 1583 cm^− 1^ showed COO stretching in tween 20. The strong and sharp band at 1082 cm^− 1^ corresponded to C-O stretching. The hydrogen bonds between tween 20, the EO, CMC, and water increase the degree of polarization of chemical bonds due to physical cross-linking. All the other characteristic bands appear in the spectra of EO and blank. So, it could be confirmed that successful loading of the EO in the nanogel.

### **Repellency effects of the nanogel containing the*Acroptilon repens’*EO**

The protection times of the nanogel, blank gel, and DEET against *An. stephensi* are shown in Fig. 1D. The efficacy of the nanogel with 310 ± 45 min was significantly (*P* = 0.005) more potent than DEET with 160 ± 17 min. Besides, the blank gel did not show any repellent characteristics.

## Discussions

*Anopheles stephensi* is one of the main malaria vectors in India, Iran, Pakistan, and the Arabian Peninsula [18]. Although using plants as repellents has been common for a long time, their effectiveness is less than synthetic types [19, 20]. Preparing nanostructures containing EOs has recently been introduced as a promising approach for meeting the challenges [21, 22]. This study proposed a nanogel containing *A. repens* EO as a natural repellent. Among the identified components, α-copaene and caryophyllene oxide each comprised more than 10% of the components of *A. repens* EO. Alph-copaene and caryophyllene oxide are two naturally occurring compounds in many EOs; they possess many biological effects such as sedative, antioxidant, anti-inflammatory, and antimicrobial properties [23, 24]. Recent research has unveiled their potential as potent tools in the battle against mosquito-borne diseases. They exhibit significant larvicidal properties and are powerful repellents, discouraging adult mosquitoes from seeking human hosts [25–27]. EOs are a mixture of compounds whose properties are a function of their constituents; however, whether the effects of the components are synergistic, antagonistic, or additive should be investigated separately.

Furthermore, the efficacy of the prepared nanogel (310 ± 45 min) was more potent than DEET with a protection time of 160 ± 17 min. This efficacy is comparable with the available reports. For instance, nanogel containing *Elettaria cardamomum* EO (2.5%) showed 63 ± 15 min protection time against *An. stephensi* [28]. Another introduced solid lipid nanoparticles containing *Zataria multiflora* EO (1%) with a protection time of 93 ± 5 min compared with non-formulated *Z. multiflora* EO with 29 ± 2 min protection time against *An. stephensi* [29]. Moreover, a nanoliposomal gel containing *Cinnamomum zeylanicum* EO showed proper repellent against *An. stephensi*, 303 ± 10 min [30]. A nanoemulsion containing *Eucalyptus globulus* with 351 min against *An. stephensi* was also reported [31]. Considering the high amount of EO (50%) in the mentioned nanoemulsion, it can be said that it is not a suitable form for developing a repellent. Due to its low viscosity, a large amount of EO evaporates or oxidizes when air is exposed [32]. Recently, CMC-based nanogel has received more attention due to mechanical resistance, viscous properties, low cost, excellent stability, and high capacity for loading EO [33, 34]. As in this study, primary nanoemulsion was gellified using CMC as thickening agent.

## Conclusion

The protection time of nanogel (310 ± 45 min) was significantly longer than DEET as a gold-standard repellent (160 ± 17 min). Therefore, it could be considered for further investigation against other mosquitoes.

### Limitations

The efficacy of the nanogel could be investigated against other mosquitoes. Besides, to provide the same conditions to reduce research bias for all bioassays, knowing that the process of doing the test was very long, we decided that one volunteer did all the samples.

### Electronic supplementary material

Below is the link to the electronic supplementary material.

Supplementary Material 1

Supplementary Material 2

## Data Availability

All data generated or analyzed during this study are included in this published article.

## Abbreviations

ATR-FTIR: Attenuated Total Reflection-Fourier Transform InfraRed
CMC: Carboxymethyl cellulose
DEET: N, N-diethyl-meta-toluamide
EO: Essential oil
