# Supplementary material for: Repellent efficacy of the nanogel containing Acroptilon repens essential oil in comparison with DEET against Anopheles stephensi
Source: BMC Res Notes. 2023 Oct 9;16:261. doi: 10.1186/s13104-023-06538-1 (PMC10561488; doi:10.1186/s13104-023-06538-1)
Supplement: Supplementary file 1 — Supplementary Material 1 [file 13104_2023_6538_MOESM1_ESM.docx]

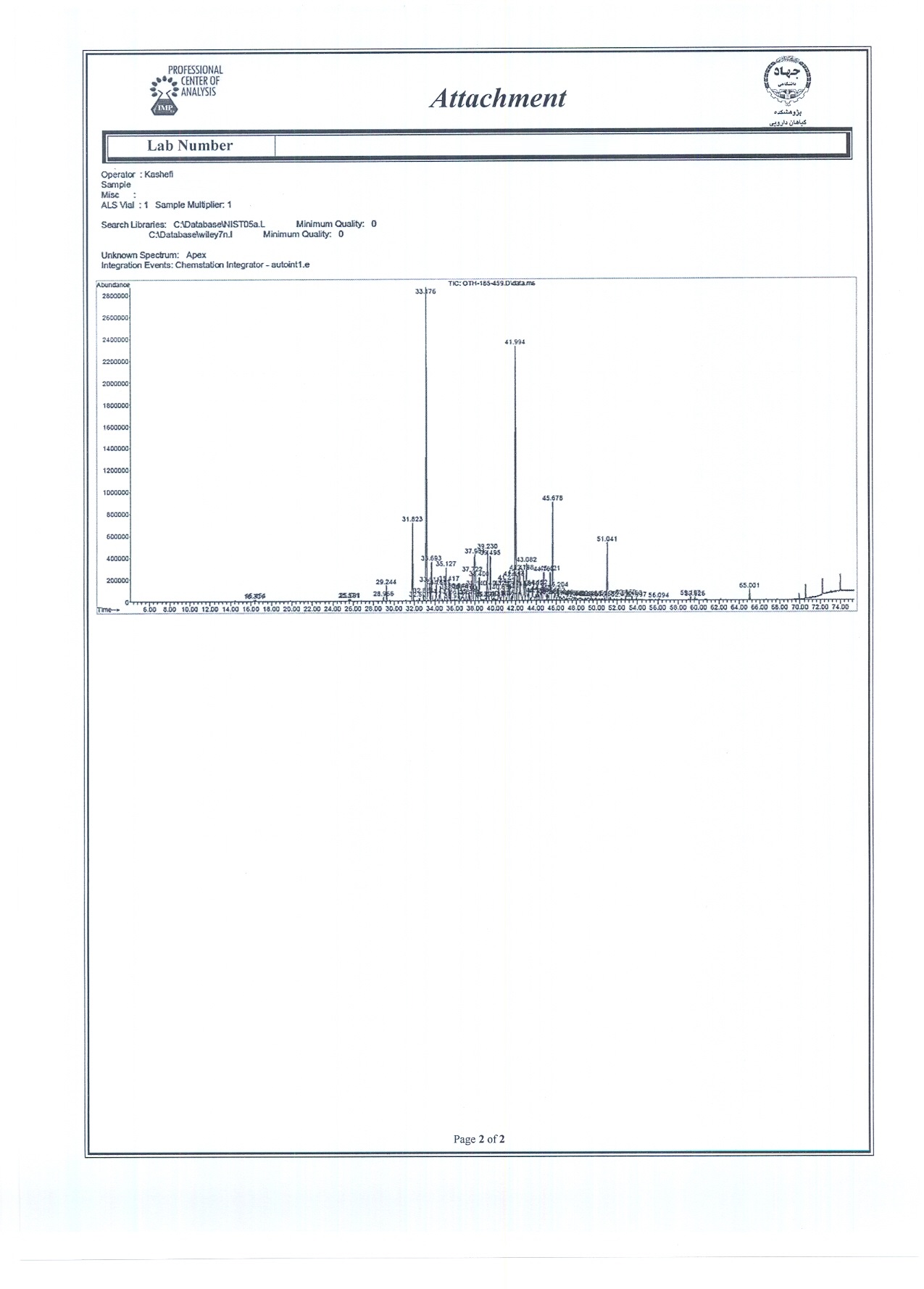


**S1: Gas chromatography-mass spectrometry recorded spectra for the identification of compounds in *Acroptilon repens* essential oil**
