# Supplementary material for: Repellent efficacy of the nanogel containing Acroptilon repens essential oil in comparison with DEET against Anopheles stephensi
Source: BMC Res Notes. 2023 Oct 9;16:261. doi: 10.1186/s13104-023-06538-1 (PMC10561488; doi:10.1186/s13104-023-06538-1)
Supplement: Supplementary file 2 — Supplementary Material 2 [file 13104_2023_6538_MOESM2_ESM.docx]

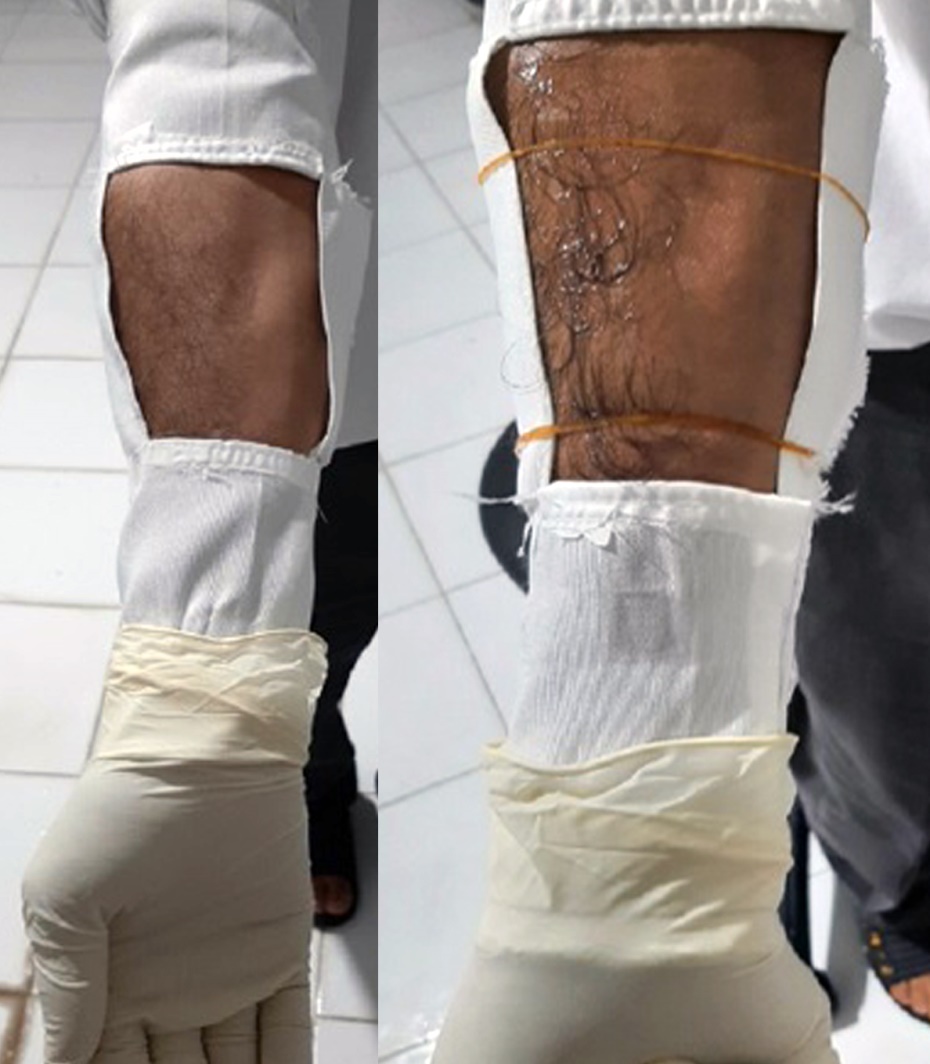


S2: Left: an exposed area with mosquitoes before treatment, Right: exposed area after treatment with nanogel or DEET
